# Identification of N-Glycoproteins of Knee Cartilage from Adult Osteoarthritis and Kashin-Beck Disease Based on Quantitative Glycoproteomics, Compared with Normal Control Cartilage

**DOI:** 10.3390/cells11162513

**Published:** 2022-08-12

**Authors:** Jing Han, Huan Deng, Yizhen Lyu, Xiang Xiao, Yan Zhao, Jiaxin Liu, Ziwei Guo, Xuan Liu, Lichun Qiao, Hang Gao, Mikko Juhani Lammi

**Affiliations:** 1 Department of Occupational and Environmental Health, School of Public Health, Health Science Center, Xi’an Jiaotong University, Xi’an 710061, China; 2 Key Laboratory of Environment and Genes Related to Diseases, School of Public Health, Health Science Center, Xi’an Jiaotong University, Xi’an 710061, China; 3 Laboratory of Resource Biology and Biotechnology in Western China (Ministry of Education), Provincial Key Laboratory of Biotechnology, College of Life Sciences, Northwest University, Xi’an 710069, China; 4 Department of Integrative Medical Biology, Umeå University, 90187 Umeå, Sweden

**Keywords:** glycoproteins, osteoarthritis, Kashin–Beck disease, cartilage

## Abstract

Glycoproteins are involved in the development of many diseases, while the type and content of N-glycoproteins in the cartilage of osteoarthritis (OA) and Kashin–Beck disease (KBD) are still unclear. This research aims to identify N-glycoproteins in knee cartilage patients with OA and KBD compared with normal control (N) adults. The cartilage samples were collected from gender- and age-matched OA (*n* = 9), KBD (*n* = 9) patients, and N (*n* = 9) adults. Glycoproteomics and label-free liquid chromatography-tandem mass spectrometry (LC-MS/MS) obtained N-glycoproteins of KBD and OA. A total of 594 N-glycoproteins and 1146 N-glycosylation peptides were identified. The identified data were further compared and analyzed with Gene Ontology (GO), Kyoto Encyclopedia of Genes and Genomes (KEGG), and Protein–Protein Interactions (PPI). Pairwise comparison of the glycoproteins detected in the three groups showed that integrin beta-1 (ITGB1), collagen alpha-1 (II) chain (COL2A1), collagen alpha-1 (VII) chain (COL7A1), carbohydrate sulfotransferase 3 (CHST-3), carbohydrate sulfotransferase 4 (CHST-4), thrombospondin 2 (THBS2), bone morphogenetic protein 8A (BMP8A), tenascin-C (TNC), lysosome-associated membrane protein (LAMP2), and beta-glucuronidase (GUSB) were significantly differentially expressed. GO results suggested N-glycoproteins mainly belonged to protein metabolic process, single-multicellular and multicellular organism process, cell adhesion, biological adhesion, and multicellular organism development. KEGG and PPI results revealed that key N-glycoproteins were closely related to pathways for OA and KBD, such as phagosome, ECM-receptor interaction, lysosome, focal adhesion, protein digestion, and absorption. These results reflected glycoprotein expression for OA and KBD in the process of ECM degradation, material transport, cell–cell or cell–ECM interaction, and information transduction. These key significantly differentially expressed N-glycoproteins and pathways lead to the degeneration and degradation of the cartilage of OA and KBD mainly by disrupting the synthesis and catabolism of basic components of ECM and chondrocytes and interfering with the transfer of material or information. The key N-glycoproteins or pathways in this research are potential targets for pathological mechanisms and therapies of OA and KBD.

## 1. Introduction

Osteoarthritis (OA) is a chronic joint disease characterized by degenerative articular cartilage changes and secondary hyperosteogeny [1]. OA mainly occurs in adults, and its incidence increases with age, resulting in a large global population of its patients [2]. Kashin–Beck disease (KBD) is mainly prevalent in Asia and has more than 100 million people at risk [3]. KBD mostly occurs in childhood (5–13 years old), with symptoms similar to OA, such as osteophytes appearing in the later stages of KBD in adulthood. The lack of nerve, blood vessel, and lymphatic tissue makes damage or degenerative cartilage changes difficult to reverse once they occur. In the later stages of OA and KBD, the clinical treatment is usually to relieve the pain with drugs or to replace the joint with surgery, resulting in a great burden to patients and society. Although effective early treatments of cartilage damage may slow the progression of diseases and reduce the pain and economic burden of patients, the pathogenesis of OA and KBD remains unclear, causing interference in finding an effective treatment for them.

Clarifying the similarities and differences of OA and KBD’s pathogenesis will provide a valuable basis for their treatment. Overall, the most significant pathological difference between them is the massive necrosis of chondrocytes in the deep tissues of epiphyseal plate cartilage and articular cartilage in KBD patients (Figure S1). Meanwhile, some pathologic similarities of cartilage damage between them have been found, such as degradation of extracellular matrix (ECM) and loss of proteoglycan and collagen. Furthermore, genomic and proteomic studies of KBD and OA have effectively confirmed that they share key genes, proteins, and pathways primarily associated with metabolism, focal adhesion, and signal transduction in ECM [3,4,5]. Additionally, they have different abundances in key proteins, including collagens, integrin, thrombospondin 2 (THBS2), thrombospondin 4 (THBS4), etc. By identifying proteins or biomarkers in the cartilage of OA, KBD, and normal people, biological processes and signal pathways involved in significantly differentially expressed proteins or biomarkers of OA and KBD could be found and enriched. It is expected to reveal the process of pathological change and contribute to the study of the pathological mechanism of OA and KBD. In recent years, the rise of glycoproteomics technology provides a new idea for further research.

N-glycosylation is an important post-translational modification (PTM) process of proteins, forming glycoproteins and participating in various physiological and pathological processes [6,7,8,9]. Glycosylation of dentin matrix protein 1 plays a critical role in fracture healing by promoting chondrogenesis [10]. Glycoproteins in cartilage explants can affect the biomechanical response of chondrocytes to compression deformation, which may reveal the mechanism of cartilage aging [11]. What is more important, a study shows that the expression of high mannose-type N-glycans decreases in the cartilage of OA model mice [12]. Above all, glycoproteins are closely related to cartilage damage [13,14,15,16]. However, few studies have systematically revealed the types and contents of N-glycoproteins in the cartilage of OA and KBD, compared with normal control.

In order to gain an in-depth understanding of the key glycoproteins and pathways of OA and KBD, we used label-free liquid chromatography-tandem mass spectrometry (LC-MS/MS) and N-glycoproteomics to analyze the biological functions and interactions of key glycoproteins and pathways in the pathogenesis of OA and KBD in this research. This finding may provide a theoretical basis for studying the pathological mechanism of cartilage injury and potential therapeutic targets for OA and KBD.

## 2. Materials and Methods 

### 2.1. Sample Collection and Preliminary Treatment

The Medical Research Ethics Committees have approved this study of Xi’an Jiaotong University (No. 2018-263). All subjects provided signed informed consent for the study participation before the study started. In all, 27 knee cartilage specimens were collected. Detailed characteristics of subjects are shown in Table S1. Nine OA cartilage specimens were obtained from patients diagnosed based on the Western Ontario and McMaster Universities’ osteoarthritis index and X-rays from Xi’an, Shaanxi. Nine KBD cartilage specimens were obtained from patients diagnosed based on the KBD clinical diagnosis criteria (WST 207-2010) in China [17,18,19] from Linyou, Shaanxi. Nine normal control (N) cartilage specimens were from donors who had no history of knee cartilage disease and had undergone knee replacement within 24 h after accidents in Xi’an, Shaanxi. Cartilage tissue of normal controls was included after being identified as not diseased by X-ray, hematoxylin-eosin staining, and toluidine blue staining. All the cartilage specimens were taken from the same part of knee from subjects (Sampling site: full-thickness cartilage blocks with an area of about 4 mm × 4 mm were cut from the loading area in front of the medial tissue and lateral sides of the tibial plateau and the loading area in front of the medial tissue).

The cartilage samples of each three subjects in each group were pooled into one biological replicate to reduce differences due to individuals. Then, samples were homogenized after being ground with liquid nitrogen, and the powder was collected into EP tubes for the subsequent process of extracting proteins. The method of extracting proteins was referred to in a previous study [20]. The concentration of lysate was determined by the BCA kit (SK3021, Sangon Biotech, Beijing, CN), and the results are shown in Table S2. 

### 2.2. Enzymatic Hydrolysis, N-Glycopeptides Enrichment and Deglycosylation

By hydrolyzing proteins with proteases, enriching N-glycopeptides with lectins, and deglycosylation, samples for subsequent mass spectrometry identification were prepared. The experimental process mainly included the following. First, we mixed the protein lysates with dithiothreitol (DTT, BioRad, San Francisco, CA, USA) and boiled them for 5 min, then divided the mixture into 10 portions and added 200 µL urea to each portion in 0.1 M Tris-HCl (pH 8.5). The solutions were then added on the top of micro con filtration units (Vivacon^®^ 500 30,000 MWCO) and centrifuged. We added 100 μL 0.05 M iodoacetamide, 200 µL urea, and 0.1 M Tris-HCl, incubated in darkness for 30 min and centrifuged. Then, 56 µg trypsin (Sigma, Saint Louis, MO, USA) and 40 µL 100 mm NH_4_HCO_3_ buffer was added and incubated at 37 °C for 18 h before eluting the sample and using 40 µL to wash the filter twice with buffer. The enzymatic hydrolysis procedure was consistent with the method in the previous research [20,21]. We collected the filtrate after centrifugation. The results of enzymatic hydrolysis of peptide concentration are shown in Table S3. Then, we took 300 μg of the peptides transferred to the filtration units, mixed with a lectin mixture (containing concanavalin A, wheat germ lectin, and castor lectin) 120 at 50 µL for being incubated for 1 h. The lectin-bound peptides were washed by 200 μL buffer five times and 50 μL 40 mM NH_4_HCO_3_ in H_2_^18^O twice. Next, 2 μg PNGase F (1 U/μL H_2_^18^O) and 50 μL 25 mM NH_4_HCO_3_ in H_2_^18^O was added in the filtrate, then was incubated at 37 °C for 3 h to perform the deglycosylation procedure. Finally, a 3 μL sample was loaded for label-free LC-MS/MS analysis.

### 2.3. Label-Free LC-MS/MS Analysis and Procession of Identification Data 

The samples were further detected and analyzed by label-free LC-MS/MS technology. Specifically, the sample was separated by Easy-nLC1200 (Thermo Scientific, Shanghai, China), a high-performance liquid chromatography nano (HPLC) system. The peptide traps Thermo Scientific EASY column (2 cm × 100 μm 5 μm-C18) and Thermo Scientific EASY column (75 μm × 100 mm 3 μm-C18) were applied to separate peptides. The separated peptides were further analyzed by Fusion Lumos mass spectrometer (Thermo Fisher Scientific, Frankfurt, Germany). Procedure details and specific parameters of label-free LC-MS/MS analysis were consistent with the previous research [21]. The label-free quantitative strategy was used to analyze the mass spectrum data generated in the identification process, compare the signal intensity of the corresponding peptides in different samples, such as mass charge ratio, and relatively quantify the N-glycopeptides achieve the purpose of analyzing the content of various N-glycopeptides in different samples. 

Further, Maxquant (version 1.6.5.0) software was used for database search and analysis to implement the identification process. The original data detected by LC-MS/MS was imported into Maxquant software for database search, and the automatically created database was uniprot-human_20190102_177661. fasta. Then, we compared with the database uniprot-human_20190102_177661.fasta to obtain information on N-glycoproteins, N-glycopeptides, and N-glycosylation sites. The false discovery rate (FDR) was set at 1% at both peptide and locus levels (FDR was calculated according to the number of matching maps obtained by retrieving the target database (the target database was the human protein database in this study), and the database obtained from our LC-MS/MS experiment (uniport-human_20190102_177661.fasta). FDR ≤ 0.01 is a recognized screening standard for omics data. So, the qualitative screening standard of glycoproteins and glycopeptides in this research was FDR ≤ 0.01. 

This study had three sample groups, including N, OA, and KBD. Based on the quantitative N-glycopeptides information, we screened out the data three repeat meeting two or more than two non-zero mass spectrum peak areas of the data in the same group. Then, we calculated the same kind of N-glycopeptides mass spectrum peak area of average in different groups. Fold-change (FC) calculations were performed for the mean value in OA vs. N, KBD vs. N, KBD vs. OA, respectively. Statistical analysis was conducted using SPSS 22.0 (SPSS Inc., Chicago, IL, USA). The data satisfies the normal distribution. Differences were analyzed using Student’s *t*-test. *p* < 0.05 were considered statistically significant differences. N-glycopeptides were screened according to the standard of FC ≥ 2 or ≤ 0.5 and *p* ≤ 0.05. PLogo (PLogo. Available online: http://plogo.uconn.edu/ (accessed on 6 February 2021)) was employed to visualize the sequence motifs around the N-glycosylation sites (5 amino acids on each side) [22,23].

### 2.4. Bioinformatics Analysis

Bioinformatics analyses were conducted to evaluate the function, pathway, or interaction network of glycoproteins identified in cartilage. Gene Ontology (GO) and Kyoto Encyclopedia of Genes and Genomes (KEGG) pathway enrichment analyses were carried out for genes annotation and visualization by the online tool (Bioinformatics. Available online: http://www.bioinformatics.com.cn/login/ (accessed on 8 February 2021)), package ggplot2 (version 3.4.0) in the R software (version 4.0.3). *p* < 0.05 was set as the cutoff criterion. In order to reveal the organization principle of functional protein network and provide new insights into protein functions, Protein–Protein Interactions (PPI) network was established by Search Tool for the Retrieval of Interacting Genes (STRING. Available online: http://string-db.org/ (accessed on 8 February 2021)) for further analysis of the differentially expressed glycoproteins. Cytoscape 3.8.2 was used for visualization. ClueGO plugin was then used to extract hub proteins from the PPI network.

## 3. Result

### 3.1. Basic N-Glycoproteomics Identification Data of N, OA, and KBD Knee Cartilages 

**Figure 1:**
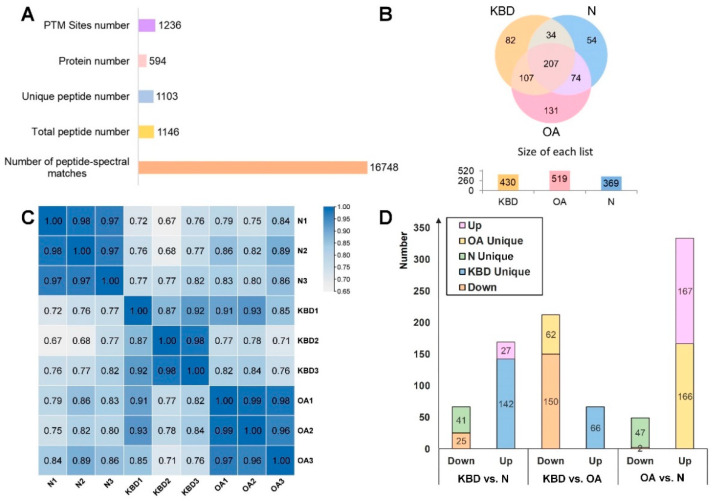
Basic identification information. (**A**) Mass spectrometric identification information. (**B**) The Venn diagram of N-glycopeptides identified in the cartilage of N, OA, and KBD group. (**C**) Correlation analysis of quantitative data. Within the N, OA, and KBD groups, respectively, the similarities within each group were high (0.87–1.00) while similarities among the three groups were low. (**D**) Modified histogram of N-glycopeptides in N, OA, and KBD group.

A total of 594 N-glycoproteins, 1146 N-glycopeptides, and 1236 N-glycosylation sites were identified (Figure 1A, Tables S4–S6). From the Venn diagram, 207 of these N-glycoproteins were commonly identified in OA, KBD, and N. Importantly, 131, 82, and 54 N-glycoproteins were specifically identified in OA, KBD, and N, respectively (Figure 1B). Correlation analysis showed high similarity within each group (0.87–1.00) and high difference among the three groups (Figure 1C). Further comparison was made based on N-glycopeptides between the two groups (Figure 1D, Table S7). N-glycopeptides with fold-change (FC) ≥ 2.0 and *p* < 0.05 were considered as up-regulated N-glycopeptides, and those with FC ≤ 0.5 and *p* < 0.05 as down-regulated N-glycopeptides. Following this criterion, when the N group was used as the control, 333 N-glycopeptides were up-regulated in OA, corresponding to 244 N-glycoproteins, and 49 N-glycopeptides were down-regulated, corresponding to 47 N-glycoproteins. In KBD, 169 N-glycopeptides were up-regulated, corresponding to 122 N-glycoproteins, while 66 N-glycopeptides were down-regulated, corresponding to 54 N-glycoproteins. When OA was used as the control, 66 N-glycopeptides were up-regulated in KBD, corresponding to 61 N-glycoproteins, and 212 N-glycopeptides were down-regulated, corresponding to 152 N-glycoproteins (Figure 1D). From the basic identification information, the number of common and unique N-glycopeptides in the three groups cartilage samples was clarified.

### 3.2. Heatmap, Volcanic Plot, and Motif Analysis Results of N-Glycopeptides

**Figure 2:**
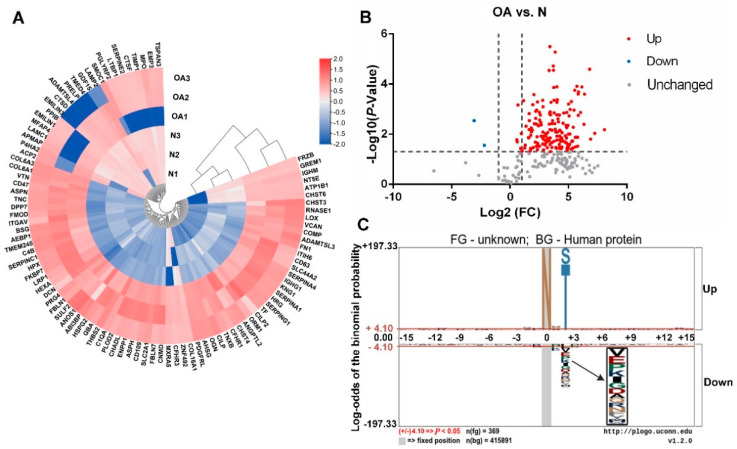
Heatmap, volcanic plot, and motif analysis in OA vs. N. (**A**) Heatmap of differentially expressed N-glycoproteins of knee cartilage in OA vs. N. Circular heatmap obviously classified samples into two distinct groups by clustering analysis. The comparative analysis aimed to determine whether significant differences could be found in OA vs. N. Within OA1, OA2, OA3, and N1, N2, N3, respectively, the differences were small. The overall difference in OA vs. N was significant. (**B**) Differential expression of N-glycopeptides from knee cartilage in OA vs. N. N-glycopeptides with FC ≥ 2.0 and *p* < 0.05 were considered as up-regulated N-glycopeptides, and those with FC ≤ 0.5 and *p* < 0.05 as down-regulated N-glycopeptides. Significantly, there were 167 up- and 2 down-regulated N-glycopeptides in OA vs. N. (**C**) Motif analysis of N-glycosylation modification sites in OA vs. N. Plogo-generated relative frequency plots of the significant sequence motif. It is reliable when the heights of amino acid residues are above 4.10 (that is to say when *p* < 0.05, the results are reliable).

**Figure 3:**
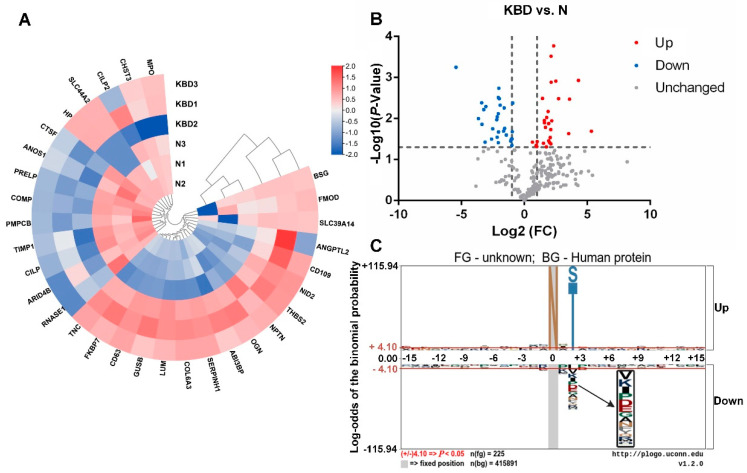
Heatmap, volcanic plot, and motif analysis in KBD vs. N. (**A**) Heatmap of differentially expressed N-glycoproteins of knee cartilage in KBD vs. N. Circular heatmap obviously classified samples into two distinct groups by clustering analysis. This is aimed to determine whether there were significant differences in KBD vs. N. Within KBD1, KBD2, KBD3, and N1, N2, N3, respectively, the differences were small. The overall difference in KBD vs. N was significant. (**B**) Differential expression of N-glycopeptides from knee cartilage in KBD vs. N. N-glycopeptides with FC ≥ 2.0 and *p* < 0.05 were considered as up-regulated N-glycopeptides, and those with FC ≤ 0.5 and *p* < 0.05 as down-regulated N-glycopeptides. There were 31 up- and 29 down-regulated N-glycopeptides in KBD vs. N. (**C**) Motif analysis of N-glycosylation modification sites in KBD vs. N. Plogo-generated relative frequency plots of the significant sequence motif. It is reliable when the heights of amino acid residues are above 4.10 (that is to say when *p* < 0.05, the results are reliable).

**Figure 4:**
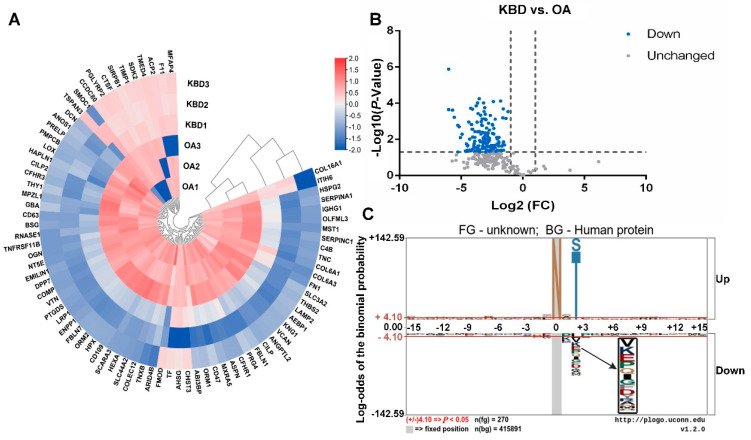
Heatmap, volcanic plot, and motif analysis in KBD vs. OA. (**A**) Heatmap of differentially expressed N-glycoproteins of knee cartilage in KBD vs. OA. Circular heatmap obviously classified samples into two distinct groups by clustering analysis. The comparative analysis aimed to determine whether significant differences could be found in KBD vs. OA. Within KBD1, KBD2, KBD3, and OA1, OA2, OA3, respectively, the differences were small. The overall difference in OA vs. KBD was significant. (**B**) Differential expression of N-glycopeptides from knee cartilage in KBD vs. OA. N-glycopeptides with fold-change (FC) ≥ 2.0 and *p* < 0.05 were considered as up-regulated N-glycopeptides, and those with FC ≤ 0.5 and *p* < 0.05 as down-regulated N-glycopeptides. Significantly, 150 N-glycopeptides were all down-regulated in KBD vs. OA. (**C**) Motif analysis of N-glycosylation modification sites in KBD vs. OA. Plogo-generated relative frequency plots of the significant sequence motif. It is reliable when the heights of amino acid residues are above 4.10 (that is to say when *p* < 0.05, the results are reliable).

Further comparison between every two groups was made by heatmap, volcanic plot, and motif analysis basing on the FC of N-glycopeptides, respectively. The results were shown in Figure 2 (OA vs. N), Figure 3 (KBD vs. N), and Figure 4 (KBD vs. OA). 

For OA vs. N, heatmap and volcanic plot showed there were 169 significantly differentially expressed N-glycopeptides, including 167 up-regulated and 2 down-regulated (Figure 2A,B). Most of the N-glycopeptides significantly differentially expressed in OA group were up-regulated compared with N group.

For KBD vs. N, there were 52 significantly differentially expressed N-glycopeptides, including 27 up-regulated and 25 down-regulated in KBD (Figure 3A,B). About half of the significantly differentially expressed N-glycopeptides in KBD group were up- and down-regulated, respectively, compared with N group.

For KBD vs. OA, there were 150 N-glycopeptides, which were all down-regulated in KBD (Figure 4A,B). Most of the N-glycopeptides significantly differentially expressed in KBD group were down-regulated compared with N group.

In comparative analysis of the three pairs, motif analysis showed that the basic amino acid structure skeleton of main glycosylation modification site of glycoproteins is N-X-S/T (X: any amino acid except proline) (Figure 2C, Figure 3C and Figure 4C). Some other significant modifications at the second amino acid residue on the left are E, I, K, P, V, etc. That is to say, most of the amino acid framework of N-glycosylated proteins in cartilage of N, OA, and KBD is N-X-S/T.

### 3.3. Bioinformatics Analysis Results of Differentially Expressed N-Glycoproteins between Every Two Groups

**Figure 5:**
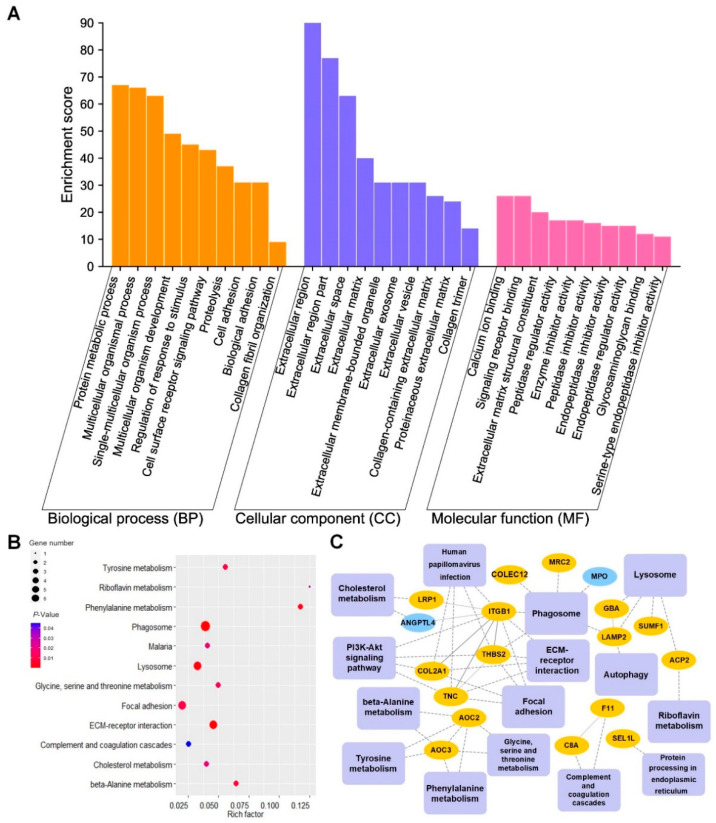
GO, KEGG pathway, and PPI analysis results of differentially expressed N-glycoproteins in OA vs. N. (**A**) GO analysis results of the differentially expressed N-glycoproteins of cartilage in OA vs. N. (**B**) Bubble diagram of KEGG pathways in OA vs. N. The blue to red gradient of bubbles of KEGG pathways represent the significance from low to high, respectively. Bubble size represents the number of differentially expressed N-glycoproteins in the KEGG pathway. (**C**) PPI network of the significantly differential N-glycoproteins and pathways in OA vs. N. Rectangles represent KEGG pathways, and circles represent N-glycoproteins. Solid lines mean the relationship has been verified by research, dashed lines mean the relationship have not been verified. The yellow and blue colors of the N-glycoproteins represent up-regulated and down-regulated, respectively.

**Figure 6:**
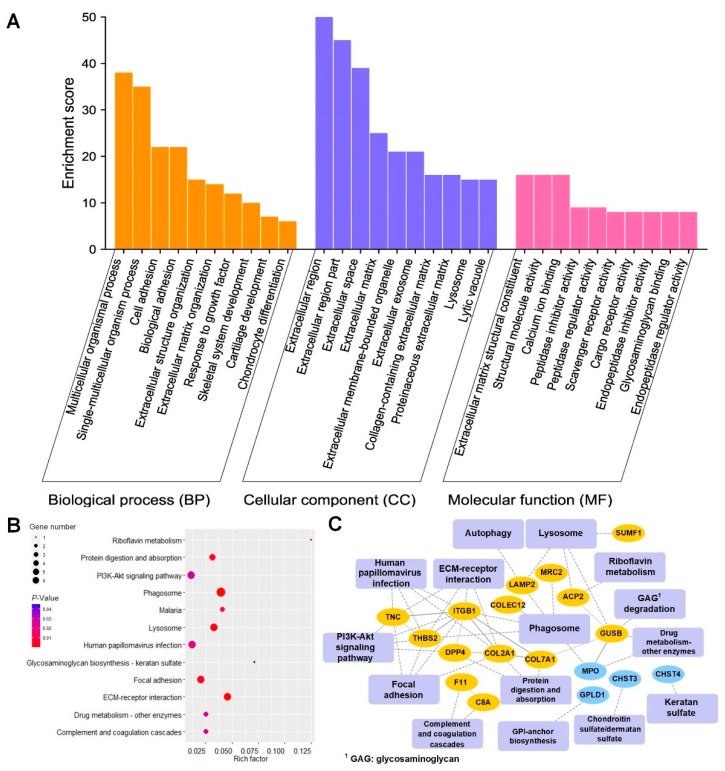
GO, KEGG pathway, and PPI analysis results of differentially expressed N-glycoproteins in KBD vs. N. (**A**) GO analysis of the differentially expressed N-glycoproteins of cartilage in KBD vs. N. (**B**) Bubble diagram of KEGG pathways in KBD vs. N. The blue to red gradient of bubbles of KEGG pathways represents the significance from low to high, respectively. Bubble size represents the number of differentially expressed N-glycoproteins in the KEGG pathway. (**C**) PPI network between KBD and N. Rectangles represent KEGG pathways, and circles represent N-glycoproteins. Solid lines mean the relationship has been verified by research, dashed lines mean the relationship has not been verified. The yellow and blue colors of the N-glycoproteins represent up-regulated and down-regulated, respectively.

**Figure 7:**
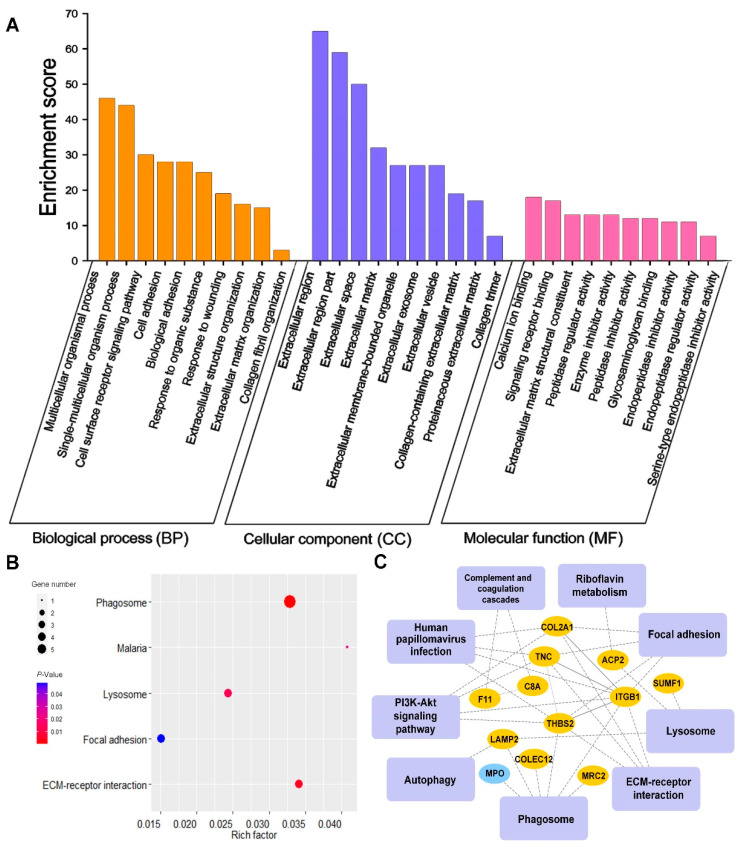
GO, KEGG pathway, and PPI analysis results of differentially expressed N-glycoproteins in OA vs. N ∩ KBD vs. N. (**A**) GO analysis of the differentially expressed N-glycoproteins of cartilage in OA vs. N ∩ KBD vs. N. (**B**) Bubble diagram of KEGG pathways in OA vs. N ∩ KBD vs. N. The blue to red gradient of bubbles of KEGG pathways represents the significance from low to high, respectively. Bubble size represents the number of differentially expressed N-glycoproteins in the KEGG pathway. (**C**) PPI network commonly shared between OA vs. N and KBD vs. N. Rectangles represent KEGG pathways, and circles represent N-glycoproteins. Solid lines mean the relationship has been verified by research, dashed lines mean the relationship have not been verified. The yellow and blue colors of the N-glycoproteins represent up-regulated and down-regulated, respectively.

Bioinformatics analysis results of differentially expressed N-glycoproteins or pathways in OA and KBD when N was used as the control were shown in Figure 5 (OA vs. N) and Figure 6 (KBD vs. N). Based on the analysis results in Figure 5 and Figure 6, we further screened out significantly differentially expressed or enriched pathways and proteins shared commonly by OA vs. N and KBD vs. N; the results were shown in Figure 7 (OA vs. N ∩ KBD vs. N).

#### 3.3.1. GO Analysis Results of Differentially Expressed N-Glycoproteins between Every Two Groups

GO analysis results, shown in bar charts, revealed the major biological process (BP), cellular component (CC), and molecular function (MF) involved in the differentially expressed N-glycoproteins (Figure 5A, Figure 6A and Figure 7A). In OA vs. N, these differentially expressed N-glycoproteins were mainly involved in protein metabolic processes, multicellular organismal processes, single-multicellular organism processes, etc. (BP in Figure 5A). In KBD vs. N, biological processes were mainly involved multicellular organismal processes, single-multicellular organism processes, cell adhesion, biological adhesion, etc. (BP in Figure 6A). In OA vs. N ∩ KBD vs. N, these differentially expressed N-glycoproteins were mainly involved in biological processes of multicellular organismal processes, single-multicellular organism processes, and cell surface receptor signaling pathway (BP in Figure 7A). Significantly differentially expressed N-glycoproteins were all mainly localized in the extracellular region, extracellular region part, extracellular space, etc. (CC in Figure 5A, Figure 6A and Figure 7A). The main molecular functions of these N-glycoproteins in OA vs. N included calcium ion binding, signal receptor binding, ECM structural constituent, etc. (MF in Figure 5A). The molecular functions of these N-glycoproteins in KBD vs. N included ECM structural constituent, structural molecule activity, calcium ion binding, etc. (MF in Figure 6A). The molecular functions of these N-glycoproteins in OA vs. N ∩ KBD vs. N included calcium ion binding, signaling receptor binding, and extracellular matrix structural constituent (MF in Figure 7A). On the whole, GO analysis revealed the main BP, CC, and MF, which were involved in the differentially expressed N-glycoproteins. The main BP included protein metabolic process, multicellular organismal process, single-multicellular organism process, cell adhesion, biological adhesion, and multicellular organism development. The main CC included extracellular region, extracellular region part, and extracellular space. The main molecular functions included calcium ion binding, signal receptor binding, ECM structural constituent, structural molecule activity, calcium ion binding, and signaling receptor binding. 

#### 3.3.2. KEGG Pathways of Differentially Expressed N-Glycoproteins between Every Two Groups

Significantly enriched KEGG pathways (*p* < 0.05) were displayed through bubble charts. In OA vs. N, KBD vs. N, and OA vs. N ∩ KBD vs. N, the top three significant KEGG pathways were all phagosome, ECM-receptor interaction, and lysosome (Figure 5B, Figure 6B and Figure 7B). In addition, there were some pathways worth paying attention to, for example, focal adhesion in OA vs. N (Figure 5B), protein digestion and absorption, and focal adhesion in KBD vs. N (Figure 6B). The above results reflected these enriched KEGG pathways mainly included phagosome, ECM-receptor interaction, lysosome, focal adhesion, protein digestion, and absorption.

#### 3.3.3. PPI Networks of Differentially Expressed N-Glycoproteins between Every Two Groups

Complex interaction relations between differentially expressed or enriched glycoproteins and glycoproteins or with KEGG pathways were displayed by PPI network diagrams. In OA vs. N, PPI network contained 15 statistically significant pathways, 18 N-glycoproteins, and 50 interactions. In total, 16 and 2 N-glycoproteins were up- and down-regulated, respectively. With 5 interactions, integrin beta-1 (ITGB1) was also found to interact with most other N-glycoproteins and pathways, followed by tenascin-C (TNC) and THBS2 (Figure 5C). In KBD vs. N, PPI network contained 15 pathways, 18 N-glycoproteins, and 50 interactions. A total of 14 and 4 N-glycoproteins were up- and down-regulated, respectively. ITGB1 was the most interacted portion, which interacted with five N-glycoproteins, followed by collagen alpha-1(VII) chain (COL7A1) and beta-glucuronidase (GUSB) (Figure 6C). In OA vs. N ∩ KBD vs. N, the PPI network contained 9 pathways, 12 N-glycoproteins, and 34 interactions. A total of 11 and 1 N-glycoproteins were up- and down-regulated, respectively. ITGB1 was found to be the most interacted protein, followed by THBS2 and TNC (Figure 7C). PPI diagrams showed the interaction between the significantly differentially expressed glycoproteins and the KEGG pathway into which the differentially expressed glycoproteins are significantly enriched. PPI diagrams shown above for each comparison between the two groups were the result of integrating the GO analysis with the KEGG analysis. The PPI networks showed the complex interaction between the differentially expressed glycoproteins and glycoproteins or pathways, in which one glycoprotein, ITGB1, was found to have a prominent proportion of connections with other network nodes.

In addition, key N-glycoproteins ITGB1, collagen alpha-1 (II) chain (COL2A1), COL7A1, carbohydrate sulfotransferase 3 (CHST-3), carbohydrate sulfotransferase 4 (CHST-4), THBS2, bone morphogenetic protein 8A (BMP8A), TNC, lysosome-associated membrane protein (LAMP2), and GUSB were revealed to be closely related to cartilage metabolism or damage based on the above results (Table S8). 

## 4. Discussion

Proteins are formed through a complex series of processes, in which PTMs give proteins their unique structure and function. N-glycosylation, which means the attachment of glycans to asparagine residues of polypeptide chains in a sequence-specific manner, profoundly surpasses the richness and complexity of other PTMs. After N-glycosylation, N-glycoproteins form, which have a significant role on cellular and biological processes and development of many diseases [6,7,8,9]. In the previous research, we initially obtained differentially expressed proteins in proteomic and N-glycoproteomic analysis, then analysed the BP, CC, and MF, then compared the proteins and N-glycoproteins differentially expressed in the two omics, and conducted in-depth discussion on the differentially expressed proteins and N-glycosylation sites [21]. Based on the previous research, we added normal controls in this research, then carried out LC-MS/MS detection and N-glycoproteomics analysis based on the label-free strategy. The aim of this research was focused on the N-glycoproteins themselves. In our bioinformatics analysis, OA and KBD were firstly compared with N group for N-glycoprotein detection, then the results of the two comparisons were combined for further comparison to obtain the shared differentially expressed N-glycoproteins and KEGG pathways of these two knee cartilage diseases. 

In the previous research, several proteins and their N-glycosylation sites related to the pathological process of KBD were found, including ITGB1, LRP1, ANO6, COL1A1, MXRA5, DPP4, and CSPG4. CRLF1 and GLG1 were proposed to be all involved in the pathological processes of KBD and OA. The key proteomics and N-glycoproteomics pathways in KBD compared with OA included ECM-receptor interactions, adhesion, phagosomes, protein digestion and absorption [21]. In this research, we used N group as the comparison standard for bioinformatics analysis to screen out the differentially expressed N-glycoproteins and pathways of OA and KBD and constructed PPI interaction networks. Based on the results of these two analyses, we further screened out the pathways and N-glycoproteins that were significantly expressed or enriched in OA and KBD compared with N. This allowed the N-glycoproteins and KEGG pathways shared by these two cartilage diseases to be screened more intensively and precisely. Key N-glycoproteins (ITGB1, COL2A1, COL7A1, CHST-3, CHST-4, THBS2, BMP8A, TNC, LAMP2, GUSB) and pathways (ECM–receptor interaction, ECM components, phagosome, lysosome, protein digestion and absorption, focal adhesion, etc.) were identified in this research. It could be found that the key pathways revealed in this research cover the previous research, which confirmed that the above pathways play an extremely important role in the pathogenesis of OA and KBD. Only one key glycoprotein, such as ITGB1, was found to be consistent with previous studies. At the same time, many other differentially expressed N-glycoproteins in OA and KBD were screened out. Key N-glycoproteins and pathways lead to the degeneration and degradation of cartilage of OA and KBD mainly by disrupting the synthesis and catabolism of basic components of ECM and chondrocytes and interfering with the transfer of material or information. Identification of N-glycoproteins helps to elucidate mechanisms of cartilage damage in OA and KBD. Key N-glycoproteins and pathways identified in this research are mainly related to metabolism of ECM and chondrocyte. The information may provide potential value for further study on the pathological mechanism of OA and KBD.

ECM is composed of complex components such as proteoglycans and collagen, and acts as metabolic microenvironment for chondrocytes, providing them with structural, biochemical, and biomechanical support [24]. ECM–chondrocyte interactions are mainly mediated by integrins, collagen, etc. [25]. ITGB1–collagen interaction reduces chondrocyte apoptosis [26]. A reduction in proteoglycan content in OA and KBD cartilage have been observed in previous research [21,27,28,29]. In this research, KEGG pathway and PPI network revealed abnormal collagen content (COL2A1 and COL7A1) in KBD and OA cartilage. COL2A1 was obviously higher in cartilage of OA and KBD than that in N. COL7A1 was higher in cartilage of KBD than in N. In addition, some N-glycoproteins involved in the composition of ECM or in ECM metabolism, such as ITGB1, CHST-3, CHST-4, THBS2, BMP8A, and TNC, also differed in OA and KBD compared to N group. 

Integrin, a class of important transmembrane glycoproteins, which promote the interaction and transmission of signals between cells and ECM, could not be ignored in ECM function [30]. The PPI networks in this research showed the interaction between the differentially expressed N-glycoproteins and N-glycoproteins or pathways, in which ITGB1, an integrin subunit, was found to have a prominent proportion of connections with other network nodes. It occupied a very important position in the PPI networks of all comparisons; therefore, ITGB1 aroused our great concern. Previous research indicated chondrocytes lacking ITGB1 are unable to form a columnar arrangement in the growth plate cartilage [30]. ITGB1 was upregulated in both OA and KBD compared to N; however, its specific function still needs to be further verified, since there are several subunits, which can pair with integrin and bind with different ECM components. 

Collagens load and cushion mechanical stress in ECM. In this research, COL2A1 was obviously higher in OA and KBD than that in N. Mutations in the COL2A1 have been observed in chondrodysplasia [31]. The increase of COL2A1 may be due to the compensatory action of chondrocytes attempting to restore the metabolic response needed to maintain chondrocyte differentiation [32]. COL7A1 is rarely identified in previous cartilage diseases studies. A study found down-regulation of COL7A1 is one of the molecular mechanisms by which estrogen may induce and disrupt the development of cartilage in zebrafish larvae [33]. COL7A1 was higher in KBD than in N. Yet, its specific role and the effect need to be explored. 

Two enzymes related to chondroitin sulfate (CS, one basic component of ECM) metabolism [29,34], CHST-3 and CHST-4, were also identified as differentially expressed N-glycoproteins. CHST-3 is a type II transmembrane protein with six N-glycosylation sites and catalyzes the transfer of sulfate to position 6 of GalNAc residues of CS [35]. Inhibition of CHST-4 may have therapeutic benefits in inflammatory diseases [36]. CHST-3 and CHST-4 were all up-regulated in KBD compared with N. Previous studies have barely detected the association between CHST-4 and KBD. Abnormal expression of CHST-4 might be one of the potential mechanisms of KBD cartilage changes and might provide substantial insight into the difference in pathogenesis between OA and KBD. 

Some significantly differentially expressed N-glycoproteins related to deformation and self-remodeling of ECM were identified, including THBS2, BMP8A, and TNC. THBS2 mediates ECM–receptor interactions [37]. Lacking THBS2 can cause defects in the collagen fibrils and change the mineral content of bone [38]. Elimination of THBS2 in mouse osteoblasts resulted in changes in the distribution of other ECM proteins, such as collagen [39]. BMP8A also plays a role in bone formation [40]. THBS2 was higher in OA and KBD than that in N. BMP8A was up-regulated in KBD compared to OA. Concerning our results, THBS2 and BMP8A might promote the remodeling of damaged cartilage. TNC, an important N-glycoprotein in the ECM, promotes cartilage repair. The expression of TNC was increased in the early stage but decreased in the late stage of cartilage damage [41]. Additionally, we found TNC was down-regulated in the cartilage of KBD patients in the late stage, which was consistent with the previous conclusion. 

In addition, two key N-glycoproteins involved in phagosome or lysosome [42,43], LAMP2 and GUSB, were differentially expressed. LAMP2 may protect the lysosomal membrane from autodigestion, maintain acidic lysosomal environment, and adhere to the cell surface to complete inter- and intracellular signal transduction [44]. LAMP2 deficiency impairs lysosomal transport to the perinuclear region, where it fuses with autophagosomes [45,46]. GUSB is a lysosomal enzyme involved in the metabolism of glycosaminoglycans (GAGs). GAGs cannot be degraded in the absence of GUSB, resulting in mucopolysaccharide storage disease VII. Patients appear to have short stature, bone dysplasia, and other symptoms [47]. Such symptoms are consistent with KBD but not with OA. LAMP2 was significantly up-regulated in OA and KBD compared, and GUSB was significantly higher in KBD than that in N. Whether and how LAMP2 or GUSB affect OA and KBD needs to be verified in future research.

Biological procession and function of N-glycoproteins in KBD and OA were mainly summarized by GO analysis. Identified N-glycoproteins mainly belonged to the synthesis, metabolism, and adhesion processes of ECM and chondrocyte (protein metabolic process, single-multicellular and multicellular organism process (any biological process, occurring at the level of a multicellular organism, pertinent to its function), cell adhesion, biological adhesion, multicellular organism development). N-glycoproteins play an important role in the molecular functions of cartilage, such as participating in the composition of cartilage ECM, regulation of molecular activity, and binding of signal molecules to receptors (calcium ion binding, signal receptor binding, ECM structural constituent, structural molecule activity). Proteolysis is the main internal mechanism of degeneration and degradation of the cartilage [48]. The stability of cartilage matrix and chondrocytes is closely related to the above biological processes and molecular functions. These molecular functions may affect processes of cartilage synthesis and catabolism in OA and KBD.

Extracellular and intracellular signal transduction and substance exchange depends on the binding of signal receptors on membrane structure and cell adhesion. Extracellular vesicles and exosomes play an important role in cartilage as a new intercellular communication and transmission mode [49,50]. Extracellular vesicles transfer bioactive molecules to recipient cells to regulate their function and activity. Exosomes are considered to be an important way to treat cartilage diseases [51,52]. Differentially expressed N-glycoproteins were mainly located in the extracellular domain, ECM, lysosome, exosome, and extracellular vesicle. More N-glycoproteins were located in the extracellular region and ECM, which corresponded to the biological processes mentioned above and also supported our idea.

Results of KEGG and PPI analysis also identified several signal pathways worthy of investigation for OA and KBD, such as phagosome, ECM-receptor interaction, lysosome, focal adhesion, protein digestion, and absorption. Some pathways in previous research, for instance, phagosome and ECM-receptor interaction, have been found at the protein level [53]. They were also proved in our research. In PPI networks, some N-glycoproteins involved in the composition or metabolism of ECM, such as ITGB1, COL2A1, COL7A1, CHST-3, CHST-4, THBS2, BMP8A, and TNC, account for interaction with other N-glycoproteins or pathways are larger. Some N-glycoproteins in lysosomes, for example, LAMP2 and GUSB, are involved in intracellular metabolism and cellular component degradation. Overall, key N-glycoproteins are closely related to these pathways and are involved in ECM synthesis and degradation, material transport, cell–cell or cell–ECM interaction, and information transduction. Above key N-glycoproteins and pathways lead to the degeneration and degradation of cartilage of OA and KBD mainly by disrupting the synthesis and catabolism of basic components of ECM and chondrocytes and interfering with the transfer of material or information.

In conclusion, this research identified the signal pathways and N-glycoproteins in knee cartilage from OA and KBD, compared with normal control adults. Identified N-glycoproteins mainly belonged to the synthesis, metabolism, and adhesion processes of ECM and chondrocyte. Key signal pathways (such as phagosome, ECM-receptor interaction, lysosome, focal adhesion, protein digestion, and absorption) were connected by the key N-glycoproteins (such as ITGB1, COL2A1, COL7A1, CHST-3, CHST-4, THBS2, LAMP2, GUSB, BMP8A, TNC). PPI results showed key information nodes of different pathway networks, which reflected N-glycoprotein expression in the process of ECM degradation, material transport, cell–cell or cell–ECM interaction, and information transduction. These key significantly differentially expressed N-glycoproteins and pathways lead to the degeneration and degradation of cartilage of OA and KBD mainly by disrupting the synthesis and catabolism of basic components of ECM and chondrocytes and interfering with the transfer of material or information. The key N-glycoproteins or pathways in this research are potential targets for pathological mechanisms and therapies of OA and KBD. Further confirmatory research will be carried out in the future to provide more specific and precise evidence for the pathological mechanisms of OA and KBD.

## Data Availability

The datasets generated and analyzed during the current study are included in this article and its Supplementary Files.

## Supplementary Materials

The following supporting information can be downloaded at: https://www.mdpi.com/article/10.3390/cells11162513/s1, Figure S1: Clinical symptoms and pathological features of Kashin–Beck disease [54,55]; Table S1: Characteristics of experiment subjects; Table S2: The N-glycoproteins quantification results of the sample after pyrolysis; Table S3: The results of quantification of enzymatic hydrolysis peptide concentration; Table S4: The list of identified N-glycoproteins; Table S5: The list of identified N-glycopeptides; Table S6: The list of identified N-glycosylation sites; Table S7: The list of significant N-glycoproteins for analysis; Table S8: TOP relative proteins or N-glycoproteins ranked by the involved signaling pathway.
