# Supplementary material for: Identification of N-Glycoproteins of Knee Cartilage from Adult Osteoarthritis and Kashin-Beck Disease Based on Quantitative Glycoproteomics, Compared with Normal Control Cartilage
Source: Cells. 2022 Aug 12;11(16):2513. doi: 10.3390/cells11162513 (PMC9406367; doi:10.3390/cells11162513)
Supplement: Supplementary file 1 [file cells-11-02513-s001.zip › cells-1797819-supplementary.pdf]

Supplementary materials

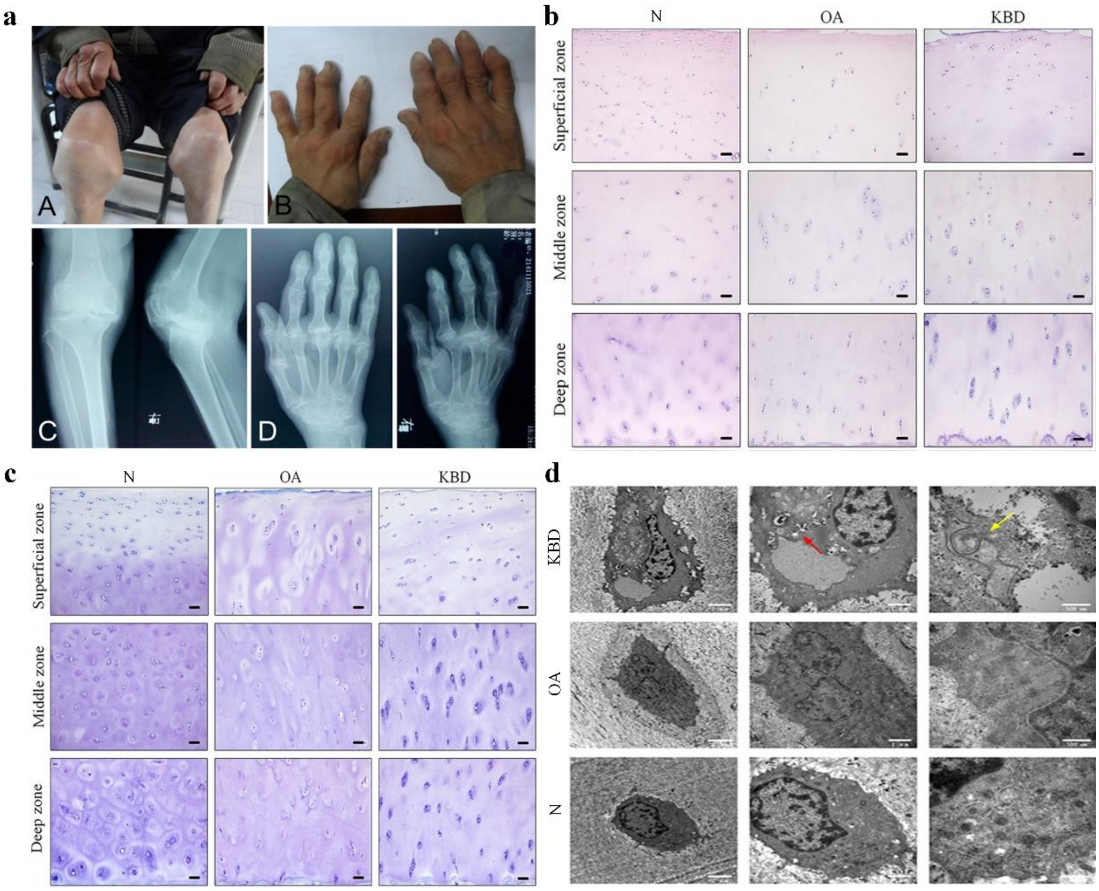

Figure S1. Clinical symptoms and pathological features of Kashin-beck disease. **a.** The main pathological features of Kashin-Beck disease (KBD) patients. Typical clinical manifestations of KBD patients [54]. **(A)** Deform joints in two knees and **(B)** flexion of the terminal finger joints or deformed fingers of grade III KBD adult. Radiographic findings in the right knee and right hand of a patient with grade II KBD. **(C)** Obvious joint space narrowing and loosened bodies in knee and D wrist joint crowded and space narrowing, phalanx thickening, and osteophyte formation. **b.** Hematoxylin and eosin (HE) staining of articular cartilage in normal (N), KBD, and osteoarthritis (OA) groups [55]. In KBD and OA groups, the cell numbers were much lower in all 3 layers compared with the N group. scale bar 20 μm. **c.** Toluidine blue (TB) staining of articular cartilage in N, KBD, and OA groups [55]. In KBD and OA groups, the rates of intensive staining areas were much lower in all 3 layers compared with the N group. scale bar 20 μm. **d.** TEM images of chondrocytes from KBD, OA patients compared with N (This part of the results has not yet been published). Compared with the N group, the KBD chondrocytes were observed mitochondrial damage (↖) and endoplasmic reticulum autophagy (↖).

Table S1. Characteristics of experiment subjects. OA: Osteoarthritis; KBD: Kashin-Beck-Disease; N: Normal adult; F: Female; M: Male. χ^2_{Gender}=0.9, F_{Age}=0.389, both *P* value > 0.05.

Sample set	KBD				OA				N			
	Sample	Subject	Age	Gender	Sample	Subject	Age	Gender	Sample	Subject	Age	Gender
		①	50	F		①	51	M		①	65	F
1	KBD1	②	58	F	OA1	②	56	F	N1	②	61	M
		③	50	M		③	68	M		③	59	M
		④	55	M		④	58	M		④	62	F
2	KBD2	⑤	61	M	OA2	⑤	60	F	N2	⑤	61	M
		⑥	66	F		⑥	57	F		⑥	57	M
		⑦	54	F		⑦	67	F		⑦	60	M
3	KBD3	⑧	68	M	OA3	⑧	55	M	N3	⑧	63	F
		⑨	67	F		⑨	61	M		⑨	60	M
	Mean		58.778	-	Mean		59.222	-	Mean		60.889	-

Table S2. The N-glycoproteins quantification results of the sample after pyrolysis. OA: Osteoarthritis; KBD: Kashin-Beck-Disease; N: Normal adult.

Sample	Weight (μg)	Volume(μL)	Concentration (μg/μL)
**KBD1**	900	120.5	7.47
**KBD2**	900	140.46	6.41
**KBD3**	900	134.63	6.68
**OA1**	900	161.23	5.58
**OA2**	900	128.71	6.99
**OA3**	900	122.25	7.36
**N1**	900	87.66	10.27
**N2**	900	82.87	10.86
**N3**	900	85.17	10.57

Table S3. The results of quantification of enzymatic hydrolysis peptide concentration. OA: osteoarthritis; KBD: Kashin-Beck-Disease; N: normal adult.

Sample	Volume(μL)	Concentration(μg/μL)	Weight (μg)
**KBD1**	200	1.609	321.8
**KBD2**	220	1.725	379.5
**KBD3**	220	1.848	406.56
**OA1**	170	2.014	342.38
**OA2**	180	1.743	313.74
**OA3**	170	2.109	358.53
**N1**	160	2.223	355.68
**N2**	160	2.104	336.64
**N3**	160	2.165	368.05

Table S4. The list of identified N-glycoproteins.

Table S5. The list of identified N-glycopeptides.

Table S6. The list of identified N-glycosylation sites.

Table S7. The list of significant N-glycoproteins for analysis.

Table S8. TOP relative proteins or N-glycoproteins ranked by the involved signaling pathway.

Uniprot Entry ID	Protein or N-glycoprotein	KBD vs OA/KBD vs N/OA vs N	Pathway name
P05556	ITGB1/Integrin beta-1	up/up/up	Phagosome; ECM-receptor interaction; Focal adhesion; Human papillomavirus infection; Cell adhesion molecules; PI3K-Akt signaling pathway; Shigellosis; Bacterial invasion of epithelial cells; Pertussis; Leishmaniasis; Arrhythmogenic right ventricular cardiomyopathy (ARVC); Hypertrophic cardiomyopathy (HCM); Small cell lung cancer; Dilated cardiomyopathy (DCM); Leukocyte transendothelial migration; Toxoplasmosis; Platelet activation; Tight junction; Axon guidance; Pathogenic Escherichia coli infection; Rap1 signaling pathway; Regulation of actin cytoskeleton; Pathways in cancer; Proteoglycans in cancer
P35442	THBS2/Thrombospondin 2	down/up/up	Phagosome; ECM-receptor interaction; Focal adhesion; Human papillomavirus infection; PI3K-Akt signaling pathway
P08236	GUSB/Beta-glucuronidase	-/up/-	Lysosome; Drug metabolism – other enzymes; Glycosaminoglycan degradation; Pentose and glucuronate interconversions Porphyrin and chlorophyll metabolism; Metabolic pathways
Q16853	AOC3/Membrane primary amine oxidase	-/-/up	Phenylalanine metabolism; beta-Alanine metabolism; Tyrosine metabolism; Glycine, serine, and threonine metabolism; Metabolic pathways
P24821	TNC/Tenascin-C	down/up/up	ECM-receptor interaction; Focal adhesion; Human papillomavirus infection; PI3K-Akt signaling pathway; MicroRNAs in cancer
P02458	COL2A1/Collagen alpha-1 (II) chain	-/up/up	ECM-receptor interaction; Protein digestion and absorption; Focal adhesion; Human papillomavirus infection; PI3K-Akt signaling pathway
P13473	LAMP2/Lysosome-associated membrane glycoprotein 2	down/up/up	Phagosome; Lysosome; Tuberculosis; Autophagy
Q7Z5Y6	BMP8A/Bone morphogenetic protein 8A	up/-/-	TGF-beta signaling pathway; Cytokine-cytokine receptor interaction; Hippo signaling pathway; Thermogenesis
P05164	MPO/ Myeloperoxidase	down/down/down/	Phagosome; Transcriptional misregulation in cancer; Acute myeloid leukemia; Drug metabolism - other enzymes
P27487	DPP4/Dipeptidyl peptidase 4	up/up/-	Protein digestion and absorption
Q02388	COL7A1/Collagen alpha-1 (VII) chain	up/up/-	Protein digestion and absorption
Q7LGC8	CHST-3/Carbohydrate sulfotransferase 3	down/down/-	Glycosaminoglycan biosynthesis - chondroitin sulfate / dermatan sulfate
Q8NCG5	CHST-4/Carbohydrate sulfotransferase 4	down/down/-	Glycosaminoglycan biosynthesis - keratan sulfate
